# Kiss1 Inhibits the Proliferation of Nasopharyngeal Carcinoma Cells *Via* Activation of the LKB1/AMPK Pathway

**DOI:** 10.3389/fonc.2021.724251

**Published:** 2022-01-18

**Authors:** Tingting Li, Yong Tian, Yixuan Wang, Zhen Cui, Zelai He, Xiao Wu, Yajun Zhang, Hao Jiang

**Affiliations:** ^1^ Department of Radiation Oncology, The First Affiliated Hospital of Bengbu Medical College, Bengbu, China; ^2^ Department of Pharmacy, The First Affiliated Hospital of Bengbu Medical Collage, Bengbu, China; ^3^ General Surgery, Po Cheung Hospital, Bozhou, China; ^4^ Department of Medical Oncology, The First Affiliated Hospital of Bengbu Medical College, Bengbu, China

**Keywords:** nasopharyngeal carcinoma, cell proliferation, KISS1, KISS1R, LKB1/AMPK pathway

## Abstract

Nasopharyngeal carcinoma (NPC) is a cancer that occurs in the nasopharynx. Infinite proliferation and distant metastasis are the main characteristics of NPC cells, and the main reason for the current failure of malignant tumor treatment. In this study, by integrating the immunohistochemical, cell transfection, western blot and real-time reverse transcriptase polymerase chain reaction (RT-PCR) analysis, we observed that the expression of KISS1 and its receptor gene (KISS1R) negatively related with the proliferation of NPC cells. Overexpression of the KISS1 genes in cells reduced cell proliferation, slow down the cell cycle, and increased apoptosis. Additionally, overexpression of these genes significantly increased Liver Kinase B1 (LKB1), phosphorylation of LKB1 and AMPK, indicated by Western blotting. Together, all of these results suggested for the first time that KISS1 and KISS1R suppress the proliferation of NPC cells by activating the LKB1/AMPK pathway, thus revealing a viable indicator for diagnosis of NPC in clinical practice.

## Introduction

Nasopharyngeal carcinoma (NPC) is a cancer that occurs in the nasopharynx, which is a common malignant tumor of the head and neck, accounting for about 78% of all head and neck cancers (1). NPC affects ∼0.025 to 0.05% of the world’s population, and the incidence of this malignancy is higher in Southern China and Southeast Asia than any other place (2). Based on the characteristics, three groups of NPC are recognized in the World Health Organization (WHO) classification: squamous cell carcinoma, differentiated cell carcinoma and undifferentiated carcinoma. NPC is difficult to detect at an early stage. That may be because the nasopharynx is not easy to examine, and the symptoms of NPC are similar to other more common symptoms. In other words, the incidence of NPC is concealed, the degree of malignancy is high, and metastasis and recurrence are prone to occur (3). At present, the main means of clinical treatment of NPC is surgery combined with radiotherapy and chemotherapy. Among them, platinum-based chemotherapy is a common treatment (4, 5), but a certain proportion of patients with advanced NPC are not ideal (6, 7). However, the proliferation of tumor cells affects the prognosis and survival of patients, so inhibiting the proliferation of tumor cells has become one of the major problems urgently solved in the field. Therefore, studying the molecular mechanism of proliferation of NPC has very important practical significance in the initiation, progression and clinical treatment of NPC.

In NPC, cancer begins in the squamous cells that line the surface of the nasopharynx. Exactly what causes the gene mutations that lead to NPC isn’t known, though factors, such as the Epstein-Barr virus, genetic susceptibility, nitrosamines consumption and tobacco smoke, which increase the risk of this cancer have been identified (8–10). A handful of genetic factors involved in the development of NPC, including immune-related HLA Class I genes, cell cycle control genes mouse double minute 2 human homolog (MDM2) and tumor protein p53 (TP53), DNA repair gene RAD51L1, cell adhesion/migration gene matrix metalloproteinase 2 (MMP2) (11), and SOX2 signaling pathway (12). However, the mechanism underlying the proliferation of NPC cells has not been clearly elucidated.

The KISS1 gene is the metastasis suppressor gene in melanoma (13), which maps to chromosome 1q32 and encodes a largely hydrophobic 145-amino-acid protein (14). The KISS1 gene product functions as tumor metastasis suppressor and is reported to act after binding with human orphan G protein-coupled receptor (hOT7T175 or GPR54, KISS1R) (15). Previous studies have shown that KISS1 and KISS1R can suppress metastasis of numerous cancers, such as breast carcinoma (16), nasopharyngeal carcinom (17), bladder (18) and ovarian cancer (19, 20). The KISS1 gene have been reported that can inhibit the proliferation and invasion of gastric carcinoma cells (21). Although the loss of KISS1 and KISS1R expression has been associated with tumor progression and poor prognosis in various cancers, the mechanism underlying this phenomenon is still unclear. However, whether the KISS1 gene and its receptor KISS1R gene are involved in the regulation and how to regulate the proliferation mechanism of nasopharyngeal carcinoma is unclear. To investigate the role of KISS1 in NPC and identify its potential mechanism in tumor proliferation will require additional experiments.

In this study, we identified that the expression of KISS1 and its receptor KISS1R in squamous cell carcinoma tissues was lower than that of nonkeratinizing squamous cell carcinoma tissues. Moreover, overexpression of the KISS1 genes in cells inhibited NPC cells proliferation, slow down the cell cycle, and increased apoptosis. Additionally, overexpression of these genes significantly increased Liver Kinase B1 (LKB1), phosphorylation of LKB1 and AMPK, indicated by Western blotting. Together, all of these results suggested that KISS1 and KISS1R inhibit the proliferation of NPC cells by activating the LKB1/AMPK pathway.

## Materials and Methods

### Patient Samples

We collected 20 NPC tissue samples, after surgical resection at the First Affiliated Hospital of Bengbu Medical College between 2015-2017 (Anhui, China). Patients who had any anticancer therapy before tissue sample collection were excluded. The tumor stage was characterized using guidelines from the 2010 American Joint Committee on Cancer and the International Union against Cancer Tumor-Node-Metastasis (TNM) classification system, and the tumor differentiation was graded according to the Edmondson and Steiner grading system. This study was approved by the Ethics Committee of the First Affiliated Hospital of Bengbu Medical College, and all patients provided written informed consent.

### Cell Culture

The subtypes of the NPC cell line, namely CNE-1 and HK-1 both with high differentiation, HNE1 and 5-8F both with low differentiation, were purchased from the Guangzhou Geneseed Biotech. Co., Ltd (Guangzhou, China). The 293T cell using for package the lentivirus was purchased from the Cell Resource Center, Shanghai Institutes for Biological Sciences, Chinese Academy of Sciences. The nasopharyngeal carcinoma cells were cultured in RPMI 1640 medium and the 293T cells were cultured in Dulbecco’s modified Eagle’s medium (DMEM, Gibco, Grand Island, USA), supplemented with antibiotics (100 U/mL penicillin and 100 mg/mL streptomycin), 2 mM L-glutamine and 10% heat-inactivated fetal bovine serum (FBS). The cells were cultured in a 37°C incubator with an atmosphere of 5% CO_2_ and 95% O_2_.

### Overexpression of the KISS1 and KISS1R Genes in 5-8F Cells

The cell line that stably expressing the KISS1R and KISS1 gene was constructed with the pCDH-CMV-GFP vector (Clontech, Mountain View, CA, USA). First, the pCDH-CMV-GFP vector containing the complementary DNA (cDNA) encoding the polypeptide of the KISS1R or KISS1, and the vectors alone were used to transfect 293T cells with pspax2 and pmd2G, then the supernatants containing kiss1, kiss1 R and control lentivirus were collected respectively 48 h later. The supernatants were applied to 5-8F cells with 5 mg/mL polybrene followed by the addition of 1 mg/mL puromycin 24 h later for screening stable cell populations expressing of the corresponding protein. Subsequently, the cells were selected and proliferated.

In all cell experiments, we used overexpression of Kiss1R and control stable cell populations and transiently transfected pcDNA3.1-kiss1 and control plasmids. For the overexpression of KISS1, 4 μg of the pcDNA3.1 plasmid containing the KISS1 cDNA sequence or a negative control were mixed with 8 μl Lipofectamine 2000 (Invitrogen, Carlsbad, CA, USA) and used to transfect the 5-8F-kiss1R cells or the 5-8F-vehicle cells for 48 h according to the manufacturer’s instructions. The cells overexpression of Kiss1R and control stable cell transiented with control plasmids referred as 5-8F-kiss1R and 5-8F-vehicle cells, transiented with pcDNA3.1-kiss1 referred as 5-8F-1R-KISS1 and 5-8F-KISS1 cells. Each experiment was replicated four times. After 48 h of the transfection, the cells were sampled for the experiments.

### EdU Staining

For 5-ethynyl-20-deoxyuridine (EdU) assay, 5-8F-kiss1R and 5-8F-vehicle cells were seeded in24-well plates at a density of 1 × 10^5^ cells per well, and the assay was carried out according to the instructions for the EdU assay kit (Beyotime). Cells were transfected with kiss1 plasmid and control plasmid for 48h and incubated for 2 h with a preheated EdU working solution (10 M) at 37°C. The cells were washed 3 times and a permeable solution was added to 24-well plates for incubating 10-15 min. After washing 3 times, 200 μl Click reaction mixture was used to incubate 30 min at room temperature in the dark. Following washing 3 times, the cell nucleus stained with DAPI for 5 minutes and washed 3 times with PBS, the images were observed and captured with a microscope.

### Proliferation Assay

The viability of cells was analyzed by the standard 3-(4, 5-dimethylthiazol-2-yl)-2, 5-diphenyltetrazolium bromide (MTT) reagent (Roche, Basel, Switzerland) assay. 5-8F-kiss1R and 5-8F-vehicle cells were seeded into 96-well plates (5×10^3^ cells/well) at 37°C with 24h. Cells were transfected with kiss1 plasmid and control for 48h. The cell culture (100 μl) and MTT solution (10 μl/well) were mixed well and added into the wells, which were incubated with 5% CO_2_ and 95% O_2_ for 4 h at 37°C. A microplate reader (Bio-Rad, USA) was used to measure the absorbance at 490 nm.

### Cell Cycle Analysis

Cells were processed as before and collected and centrifuged in a centrifuge at 600 g for 10 min. The pellet was fixed with cold 70% ethanol for 2 h at 4°C and then centrifuged at 3000 g for 5 min, followed by resuspension in 100 µl PBS and incubation with 5 µl RNAse (20 µg/ml) for 30 min at 37°C. Then the cells were stained with propidium iodide (50 µg/ml) for 30 min and counted using an Accuri™ C6 flow cytometer (BD Biosciences). The results were analyzed using ModFit S software.

### Cell Apoptosis Analysis

Cells were collected and washed twice, then resuspensed in 500 µl binding buffer. Subsequently, they were stained with 5 µl (250 µg/ml, final concentration) Annexin V mixed with 5 µl propidium iodide (1 µg/ml, BD Biosciences). Stained cells were quantified by an Accuri™ C6 flow cytometer, and the data were displayed on a graph by FlowJo software version 10 (FlowJo LLC, USA). All experiments were conducted in triplicates.

### Immunohistochemical Staining

The tissue specimens were fixed with formalin and embedded in paraffin blocks, and then sliced into 6 µm thick sections. Paraffin sections for xylene dewaxing and alcohol gradient dehydration. After antigen retrieval and blocking, the sections were incubated with KISS1 (1:200, rabbit, Thermo Fisher) and KISS1R (1:200, rabbit, CST) antibody at 4˚C overnight. Washed 3 times with PBS and were incubated with horseradish peroxidase (HRP)-labeled Goat Anti-Rabbit IgG H&L (1:200, bs-0295G) at room temperature for 1 h and washed 3 times with PBS, added one drop of DAB dropwise for staining a moment. After the color was displayed, rinsed the excess DAB with running water then the slides were counterstained with hematoxylin and mounted, then the images were visualized and captured with a microscope (Zeiss, Germany).

### Real-Time Polymerase Chain Reaction Analysis

Trizol reagent (Invitrogen) was used to extract total RNA from cells, according to instructions from the manufacturer. The purity and concentration of the isolated total RNA were measured using a UV spectrophotometry. Then, the cDNA was synthesized using a qRT-PCR kit (TransGen) and amplified in a reaction mixture containing 10 µl of master mix SYBR Green, 1 µl of each primer (**Table S1**) of KISS1, KISS1R, CCND1, CCND3, CCNE1, CCNE2, CCNA1, CCNA2, CCNB1, CCNB2, CDKN1A, CDKN1B, CDK1, CDK2, CDK4, CDK6, CHK1, CHK2, LKB1, CAMKK1, CAMKK2, AMPKα1, AMPKα2, 1 µl cDNA template, and 8 µl ddH_2_O. The mRNA expression levels were normalized to those of the housekeeping gene, glyceraldehyde 3-phosphate dehydrogenase (GAPDH). The relative gene expression was calculated with the LightCycle Application (LC96, USA).

### Western Blot

Protein lysates from cells were prepared by using a lysis buffer. The proteins were purified after centrifugation. The protein sample was separated using gel electrophoresis, and transferred to the membrane using previously a described method (22). After blocking with 5% non-fat milk on a shaking bed and then incubated with the primary antibodies against KISS1 (1:1000, rabbit, Thermo Fisher), KISS1R (D9D7C) (1:1000, rabbit, CST), LKB1 (1:1000, rabbit, CST), Phospho-LKB1 (Ser428) (C67A3) (1:1000, rabbit, CST), CaMKK2 (D8D4D) (1:1000, rabbit, CST), AMPKα (D5A2) (1:1000, rabbit, CST), Phospho-AMPKα (Thr172) (D4D6D) (1:1000, rabbit, CST) and GAPDH (1:1000, rabbit, CST) and subsequently incubated with relevant anti-rabbit IgG-HRP (1:5000, CST) secondary antibodies. Finally, the visualization of protein bands was accomplished with a gel imaging system (Fusion Solo, France). The quantification of the bands’ signals was performed using by ImageJ software.

### Illumina Transcriptome Library Preparation and Sequencing

To prepare RNA library for transcriptome sequencing, total RNA was extracted using RN38 EASY spin Plus RNA Kit (Aidlab Biotechnologies Co., Ltd., Beijing, China). The extracted RNA was first treated with RNase-free DNase I (Takara Inc., Kyoto, Japan) for 45 min at 37°C to remove residual DNA. Using gel electrophoresis and spectrophotometry (Quawell Q5000, San Jose, CA, USA) to assess the quantity and quality of isolated RNA. In addition, using RNA Nano 6000 assay Kit from Agilent Bioanalyzer 2100 system (Agilent Technologies, CA, USA) measured RNA integrity. Furthermore, mRNA isolation, cDNA synthesis, addition of adapters, PCR amplification and RNA-Seq was performed by the staff at Beijing Biomarker Technologies (Beijing, China). Briefly, a total of 3 mg RNA per sample was used as input material for sample preparations. Next, using NEB Next Ultra RNA Library Prep Kit from Illumina (NEB, USA) constructed the mRNA-Seq libraries. The mRNA was purified using oligo (dT) magnetic beads, and then digested into fragments using NEB Next First Strand Synthesis Reaction Buffer (5X). Subsequently, the fragmented mRNA was used to synthesize first-strand cDNA by using reverse transcriptase, RNase H- and random hexamer primers. The second strand cDNA synthesize was achieved using DNA Polymerase I, RNase H, and dNTPs. Next, the PCR products were purified using AMPure XP system (Beckman Coulter, Beverly, USA) to construct cDNA libraries, which were subsequently assessed by Agilent Bioanalyzer 2100 system, and sequenced on an Illumina HiSequation 4000 platform. Finally, the paired-end reads were generated.

### ATP Measurement

The cells were collected from the 12-well plate into the centrifuge tube, the supernatant was discarded; 1mL of extract was added into tube, and the cells were crushed by ultrasonic for 1min (ice bath, 20% intensity or 200 W, ultrasonic for 2 s, stop for 1 s); 10000 g centrifugation at 4°C for 10min; then the supernatant was taken to another EP tube, and 500 μl chloroform was added to the tube for full vibration and mixing; 10000 g centrifugation at 4°C for 10 min, the supernatant was collected for detection; The preparation of reactants were conducted in standard tube and sample tube according to the instructions of ATP Content Assay Kit (Solarbio, BC0300); After the reactants were fully mixed, the light absorption value A1 at 340 nm for 10 s was determined immediately; Then the colorimetric dish was placed together with the reaction solution in a water bath at 37°C for 3 min; and the light absorption value A2 at 3 min 10 s was determined. ΔA (sample) = A2 (sample tube)-A1 (sample tube), ΔA (standard) = A2 (standard tube)-A1 (standard tube). ATP content (μmol/10^6^ cell) = 0.125 × ΔA (sample) ÷ ΔA (standard). The final calculated result is the relative content of ATP concentration.

### Animal Experiments

The animal study was reviewed and approved by the First Affiliated Hospital of Bengbu Medical College. A total of 2×10^6^ 5-8F cells (Control) (1×10^7^ cell/mL, 200 µl) and KISS1 overexpressing cells (OE-KISS1) were injected subcutaneously into dorsal neck of nude mice. After cells inoculation, tumors growth of the injected cells were extracted every other week to determine their volumes and weights. A caliper was used to measure tumor diameter, and tumor volume was calculated using the following formula: (Length × width^2^)/2 (23).

### Statistics

All the data in this study are presented as the mean ± SEM. The unpaired Student’s t-test or ANOVA (one-way or two-way) test was used to statistically analyse the experimental data between different groups, to determine if differences among groups are significant. All the analyses were performed using GraphPad Prism7 (GraphPad Inc., La Jolla, CA, USA). P values <0.05 was considered statistically significant.

## Results

### KISS1 and KISS1R Are Lowly Expressed in Poorly Differentiated Nasopharyngeal Carcinoma Cells

**Figure 1:**
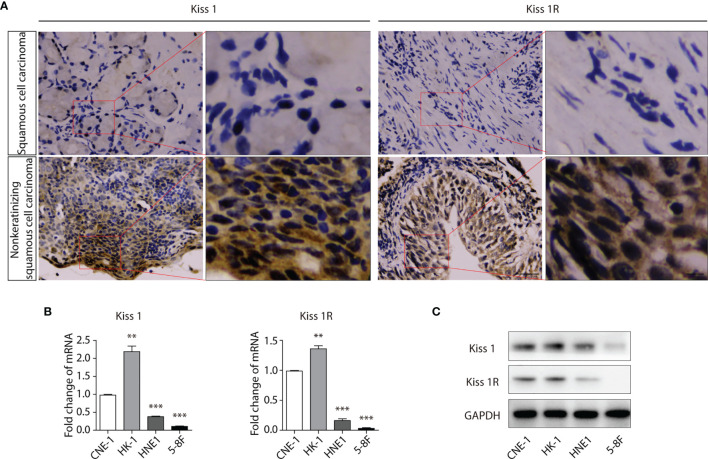
Expression of KISS1 and KISS1R gene positively with differentiation of human nasopharyngeal cancer cells. **(A)** Representative images of KISS1 and KISS1R immunostaining in squamous cell carcinoma tissues and nonkeratinizing squamous cell carcinoma tissues. Scale bar, 50 μm. **(B)** RT-PCR analysis of KISS1 and KISS1R expression in well differentiated nasopharyngeal carcinoma cells (CNE-1 cells and HK-1 cells) and in poorly differentiated nasopharyngeal carcinoma cells (HNE1 cells and 5-8F cells). **(C)** Representative bands showing KISS1 and KISS1R expression in well differentiated nasopharyngeal carcinoma cells (CNE-1 cells and HK-1 cells) and in poorly differentiated nasopharyngeal carcinoma cells (HNE1 cells and 5-8F cells) by western blot. Unpaired t test for **(B)**. Data are presented as the mean ± SEM, **P < 0.01, ***P < 0.001.

Squamous cell carcinoma is a type of human nasopharyngeal carcinoma (NPC). To investigate the expression level of KISS1 and KISS1R in human NPC tissues. The protein expression of KISS1 and KISS1R were analysed in human NPC tissues. First, we found that Kiss1 and Kiss1R were lowly expressed in squamous cell carcinoma tissues and highly expressed in nonkeratinizing squamous cell carcinoma tissues by immunohistochemistry (**Figure 1A**). The RT-PCR analysis revealed that compared with well differentiated nasopharyngeal carcinoma cells, such as CNE-1 cells and HK-1 cells, the mRNA of KISS1 and KISS1R were lowly expressed in poorly differentiated nasopharyngeal carcinoma cells, such as HNE1 cells and 5-8F cells (**Figure 1B**). The western blot protein expression analysis revealed that KISS1 and KISS1R were also lowly expressed in poorly differentiated nasopharyngeal carcinoma cells compared with well differentiated nasopharyngeal carcinoma cells (**Figure 1C**). Together, these results indicate that the expression of KISS1 and KISS1R positively associated with differentiation of human NPC tissues.

### Overexpression of KISS1 Inhibits NPC Cells Proliferation, Cell Cycle and Promotes Apoptosis *In Vitro*

**Figure 2:**
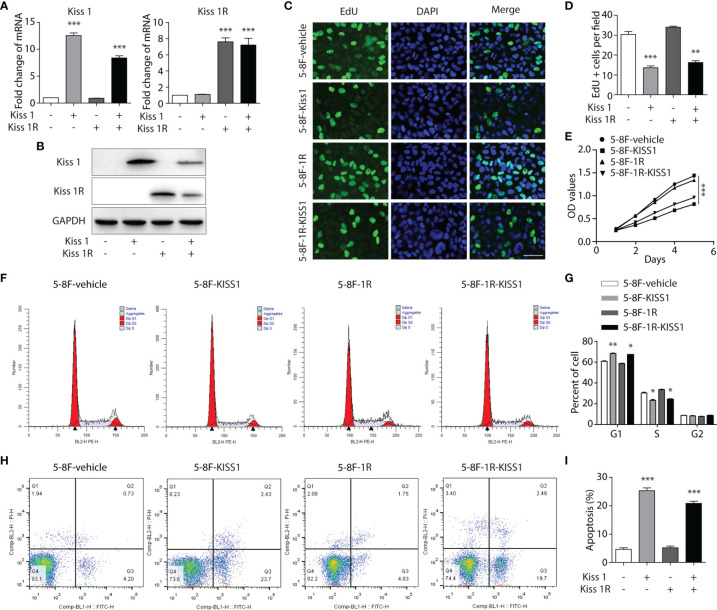
Overexpression of KISS1 effects on cell proliferation and apoptosis. **(A)** The mRNA and **(B)** protein levels of KISS1 and KISS1R were determined after 5-8F-vehicle and 5-8F-1R cells were transfected with KISS1. **(C)** Representative images of EdU stained (green) and DAPI stained nuclei (blue) in 5-8F-vehicle, 5-8F-KISS1, 5-8F-1R and 5-8F-1R-KISS1 cells. Scale bar, 50μm. **(D)** Proliferative ability data of 5-8F-KISS1, 5-8F-1R and 5-8F-1R-KISS1 cells compared with 5-8F-vehicle cells. **(E)** Cell proliferation of 5-8F-vehicle, 5-8F-KISS1, 5-8F-1R and 5-8F-1R-KISS1 cells were analyzed with MTT assays. **(F)** Cell cycle was tested and **(G)** quantified by flow cytometry in 5-8F-vehicle, 5-8F-KISS1, 5-8F-1R and 5-8F-1R-KISS1 cells. **(H)** Cell apoptosis was tested and **(I)** quantified by flow cytometry in 5-8F-vehicle, 5-8F-KISS1, 5-8F-1R an d 5-8F-1R-KISS1 cells. Unpaired *t* test for **(A, D, G, I)**; ANOVA for **(E)**. Data are presented as the mean ± SEM, *P < 0.05, **P < 0.01, ***P < 0.001.

In order to evaluate the role of KISS1 and KISS1R in the proliferation of human NPC cells, these genes were overexpressed in 5-8F cells. The 5-8F-KISS1 and 5-8F-1R cell lines that stably express the KISS1 or KISS1R alone in the 5-8F cells were generated (**Figures 2A, B**). Then, the 5-8F-1R and 5-8F-vehicle cell lines were transfected with the plasmid expressing the KISS1 gene, which called 5-8F-1R-KISS1 and 5-8F-KISS1 cells. When the level of KISS1 increased, the cell proliferation rate was significantly decreased as detected by 5-ethynyl-2’-deoxyuridine (EdU) (**Figures 2C, D**) and MTT assay (**Figure 2E**). We subsequently explored whether overexpression of KISS1 could influence the cell cycle and apoptosis of NPC cells. Flow cytometric analysis indicated that overexpression of KISS1 induced G1 cell cycle arrest, accompanied by a decrease in the number of S-phase cells (**Figures 2F, G**). We then stained the cells with annexin V-FITC and PI, and found overexpression of KISS1 promoted apoptosis of 5-8F-KISS1 and 5-8F-1R-KISS1 cells (**Figures 2H, I**). These results indicated that both inhibition of cell cycle progression and induction of apoptosis, might contribute to reduced proliferation due to overexpression of KISS1 in NPC cells.

### High-Throughput Sequencing of NPC Cells Overexpressing KISS1

**Figure 3:**
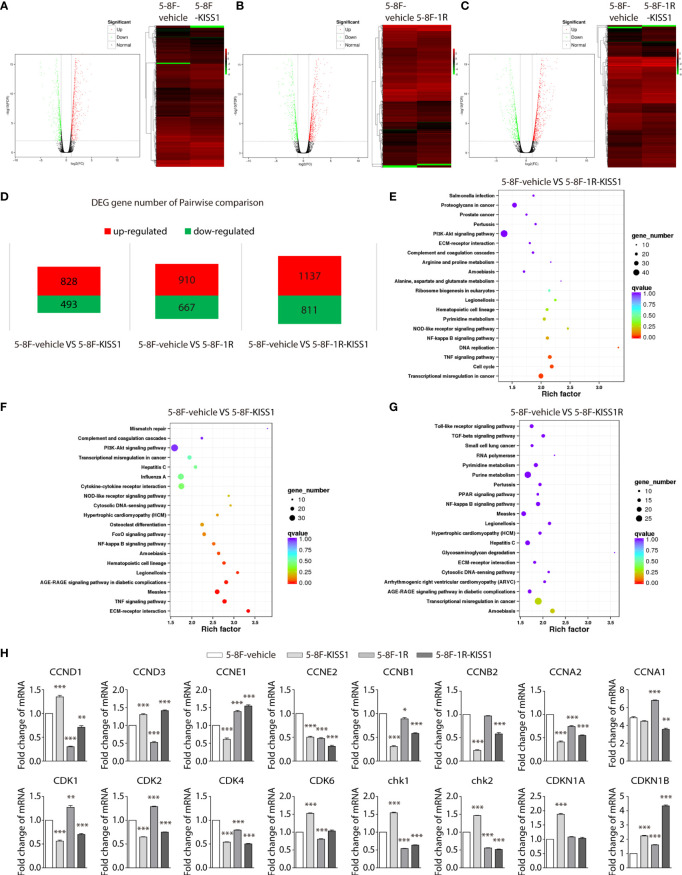
High-throughput sequencing of NPC cells overexpressing KISS1. **(A–C)** Left: volcano plot showing significantly differentiated genes between 5-8F-vehicle cells and 5-8F-KISS1 cells **(A)** 5-8F-vehicle cells and 5-8F-1R cells **(B)** 5-8F-vehicle cells and 5-8F-1R-KISS1 cells **(C)**. Right: heat map of differentially expressed genes determined by transcriptome RNA-seq of 5-8F-vehicle cells and 5-8F-KISS1 cells; **(A)** 5-8F-vehicle cells and 5-8F-1R cells **(B)** 5-8F-vehicle cells and 5-8F-1R-KISS1 cells **(C)**. Normalized z-score values (high: red; low: green) were calculated for each differentially expressed gene (row). **(D)** Number of genes that have changed in pairwise comparison. **(E)** The KEGG pathway enrichment analysis for the biological processes of differentially expressed genes in 5-8F-vehicle cells and 5-8F-1R-KISS1 cells. **(F)** The KEGG pathway enrichment analysis for the biological processes of differentially expressed genes in 5-8F-vehicle cells and 5-8F-KISS1 cells. **(G)** The KEGG pathway enrichment analysis for the biological processes of differentially expressed genes in 5-8F-vehicle cells and 5-8F-KISS1R cells. **(H)** RT-PCR verified the expression of cycle-related genes in 5-8F-vehicle cells, 5-8F-KISS1 cells, 5-8F-1R cells and 5-8F-1R-KISS1 cells. Data are presented as the mean ± SEM, *p < 0.05, **p < 0.01, ***P < 0.001.

To understand the molecular mechanism of KISS1’s role in inhibiting NPC cell growth, we performed illumina transcriptome sequencing to identify genes regulated by KISS1, KISS1R and KISS1/KISS1R in 5-8F cells. Microarray analysis of the genes expression revealed significant change in 5-8F-KISS1 cells, 5-8F-1R cells and 5-8F-1R-KISS1 cells, in comparison to 5-8F-vehicle cells (**Figures 3A–D**). Compared with the 5-8F-vehicle cells, the gene expression of 5-8F-KISS1 cells (**Figure 3A**), 5-8F-1R cells (**Figure 3B**) and 5-8F-1R-KISS1 cells (**Figure 3C**) were regulated to different degrees (**Figure 3D**). The KEGG pathway enrichment analysis of the transcriptome sequencing of the 5-8F-1R-KISS1 cells showed significant changes in transcriptional misregulation in cancer, TNF signaling pathway, DNA replication, and the cell cycle pathway compared with 5-8F-vehicle cells (**Figure 3E**); the KEGG pathway enrichment analysis of the transcriptome sequencing of the 5-8F-KISS1 cells showed significant changes in ECM-receptor interaction, TNF signaling pathway, Measles, AGE-RAGE signaling pathway in diabetic complications, legionellosis, hematopoietic cell lineage, and amoebiasis compared with 5-8F-vehicle cells (**Figure 3F**); the KEGG pathway enrichment analysis of the transcriptome sequencing of the 5-8F-KISS1R cells showed no significant changes compared with 5-8F-vehicle cells (**Figure 3G**), suggesting that KISS1/KISS1R may play an important role in the cell cycle of tumor cells. Next, the mRNA expression levels of cycle-related genes were analysed in 5-8F-vehicle, 5-8F-KISS1 cells, 5-8F-1R cells and 5-8F-1R-KISS1 cells (**Figure 3H**). We found that KISS1 and KISS1R sometimes have the same effect on gene regulation, sometimes have the opposite effect; When KISS1 and KISS1R are co-expressed, sometimes the function of KISS1 is dominant, and sometimes the function of KISS1R is dominant. These results indicated that there are similarities and differences between KISS1 and KISS1R in regulating NPC cell function.

### Activation of the LKB1/AMPK Pathway Inhibit the Cell Proliferation Caused by Expression of KISS1/KISS1R

**Figure 4:**
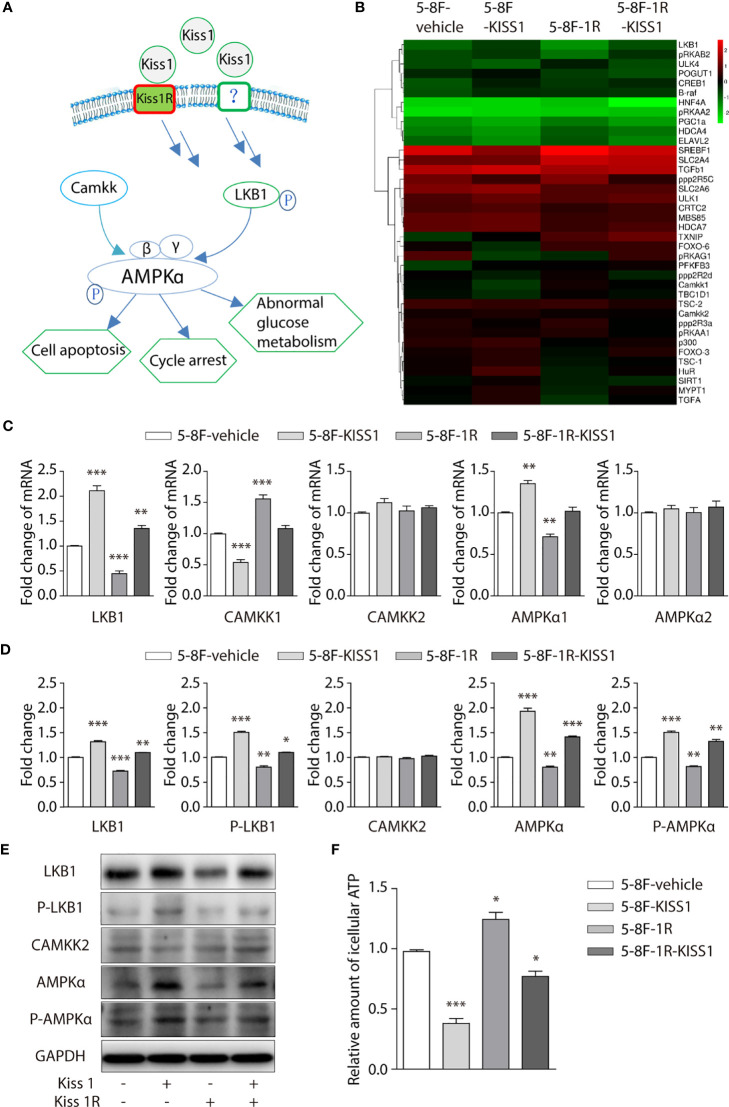
Overexpression of KISS1 increased LKB1, phosphorylation of LKB1 and AMPKα. **(A)** Schematic diagram of AMPK signaling pathway. **(B)** Heat map of gene expression related to AMPK pathway in 5-8F-vehicle cells, 5-8F-KISS1 cells, 5-8F-1R cells and 5-8F-1R-KISS1 cells. **(C)** RT-PCR verified the expression of LKB1, CAMKK1, CAMKK2, AMPKα1, AMPKα2 genes in 5-8F-vehicle cells, 5-8F-KISS1 cells, 5-8F-1R cells and 5-8F-1R-KISS1 cells. **(D)** Western blot analysis of LKB1, P-LKB1, CAMMKK2, AMPKα, P-AMPKα and GAPDH expression in 5-8F-vehicle cells, 5-8F-KISS1 cells, 5-8F-1R cells and 5-8F-1R-KISS1 cells. **(E)** Representative bands show that the expression of LKB1, P-LKB1, CAMKK2, AMPKα, P-AMPKα and GAPDH in 5-8F-vehicle cells, 5-8F-KISS1 cells, 5-8F-1R cells and 5-8F-1R-KISS1 cells. **(F)** Relative amount of icellular ATP in 5-8F-vehicle cells, 5-8F-KISS1 cells, 5-8F-1R cells and 5-8F-1R-KISS1 cells. Unpaired t test for **(C)** and **(E)**. Data are presented as the mean ± SEM, *P < 0.05, **P < 0.01, ***P < 0.001.

AMP-activated protein kinase (AMPK) signaling pathway (**Figure 4A**) plays an important role in regulating the cell cycle. In order to further investigate the underlying mechanism of KISS1 affecting cell cycle. We performed heat map analysis of gene expression related to AMPK pathway in 5-8F-vehicle cells, 5-8F-KISS1 cells, 5-8F-1R cells and 5-8F-1R-KISS1 cells, found that the expression of Liver Kinase B1 (LKB1) gene changed (**Figure 4B**). LKB1 is a tumor suppressor gene, which is the critical upstream kinase required for AMPK activation (24, 25). AMPK can be phosphorylated and activated by LKB1 and calcium dependent protein kinase kinase (CaMKK) (26, 27). Previous study showed that LKB1 phosphorylates and activates AMPK, which negatively regulates cancer cell proliferation and metabolism (28). We subsequently explored whether LKB1 or CaMKK is involved in the cell cycle regulated by KISS1/KISS1R. The RT-PCR analysis revealed that compared with 5-8F-vehicle cells, the mRNA of LKB1 and AMPKα1 were up-regulated in 5-8F-KISS1 cells and 5-8F-1R-KISS1 cells, while CAMKK1 was down-regulated (**Figure 4C**). The Western blot analysis results showed that compared with 5-8F-vehicle cells, the expression of LKB1, phosphorylated LKB1 and AMPKα were up-regulated in 5-8F-KISS1 cells and 5-8F-1R-KISS1 cells, but not the expression of CAMKK2 (**Figures 4D, E**). As an energy sensor, AMPK is activated when the intracellular AMP/ATP ratio rises (29). Therefore, we detected the level of ATP in the cells, and found that the level of ATP in 5-8F-KISS1 cells and 5-8F-1R-KISS1 cells decreased, but the ATP levels in 5-8F-1R cells increased (**Figure 4F**). These results suggest that KISS1R may partially neutralize the function of KISS1. In the absence of KISS1R, KISS1 may have other unknown mechanisms. Taken all these data together, we can conclude that KISS1/KISS1R negatively regulates the proliferation of NPC cells, which may be related to the phosphorylation of LKB1 and activation of the AMPK pathway.

### KISS1 Slow Down the Growth of NPC Cells *In Vivo*

**Figure 5:**
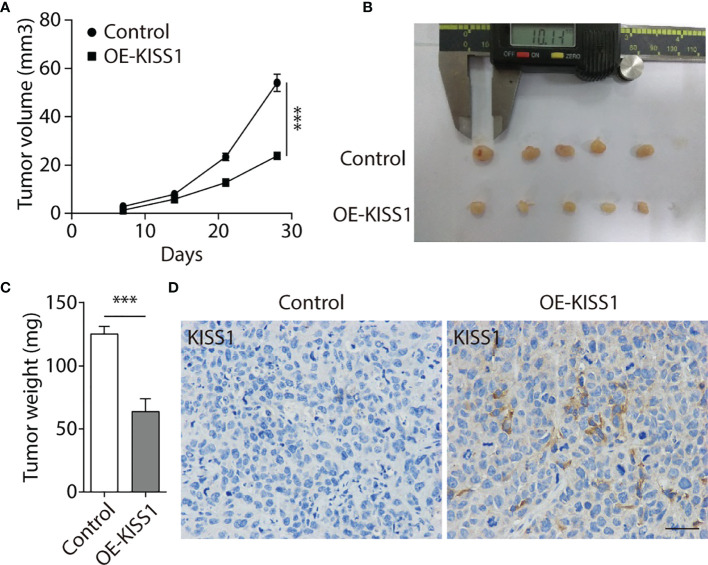
Overexpression of KISS1 suppresses tumor growth *in vivo.* **(A)** Volume quantification of subcutaneous tumors formed by 5-8F and OE-KISS1 cells every other week after 5-8F cells injection. **(B)** Images of tumors removed from nude mice every other week after injection of 5-8F and OE-KISS1 cells. **(C)** Weight quantification of extracted subcutaneous tumors formed by 5-8F and OE-KISS1 cells at the 4th week after 5-8F cells injection. **(D)** Representative immunohistochemical images of KISS1 in NPC tissues injected with 5-8F and OE-KISS1 cells. Scale bar, 50 μm. ANOVA for **(A)** unpaired t test for **(C)**. Data are presented as the mean ± SEM, ***P < 0.001.

To further explore the function of KISS1 *in vivo*, 5-8F cells infected by lentivirus vectors that could stably overexpress KISS1 gene (OE-KISS1) were subcutaneously injected into the dorsal neck regions of BALB/C nude mice for 4 weeks. We observed that mice injected with 5-8F cells overexpressing KISS1 formed smaller tumors compared with the control (**Figures 5A, B**). We then measured the weight of tumors and found the weights of KISS1 overexpressing were significantly decreased compared with control tumors (**Figure 5C**), and immunohistochemical staining showed that KISS1 overexpression was able to markedly decreased the proliferation of NPC cells (**Figure 5D**). We can conclude that KISS1 expression was negatively correlated with the tumor size. In summary, these results indicated that KISS1 gene negatively regulates the proliferation of NPC cells.

## Discussion

Infinite proliferation and distant metastasis are the main characteristics of various cancer cells, and the main reason for the current failure of malignant tumor treatment. Nasopharyngeal carcinoma is one of the representatives. The role of KISS1/KISS1R signaling is yet unclear in cancer, though it has been proposed to suppress cancer metastasis, such as melanoma, gastric cancer, breast cancer and pancreatic. Due to the lack of studies evaluating the role of KISS1 and KISS1R in the proliferation of nasopharyngeal carcinoma cells, we have tried to assess its role and molecular mechanism in nasopharyngeal carcinoma progression.

Previous works in our laboratory showed that *KISS1* gene suppresses metastasis of nasopharyngeal cancer *via* activation of the ERK1/2 pathway (17). This work showed that KISS1 and KISS1R were lowly expressed in squamous cell carcinoma tissues and highly expressed in nonkeratinizing squamous cell carcinoma tissues by immunohistochemistry. At the same time, the expression levels of KISS1 and KISS1R are positively correlated with the differentiation degree of cells in the nasopharyngeal carcinoma cell line, indicated by RT-PCR and western blotting. These data suggested that the expression of *KISS1* and *KISS1R* genes may be negatively correlated with the proliferative capacity of nasopharyngeal cancer cells. In addition, we overexpressed *KISS1* and *KISS1R* in 5-8F cells, and found that the cells overexpressed *KISS1* and the cells co-overexpressed *KISS1-KISS1R* showed a low proliferative ability, but not the cells overexpressed *KISS1R*. This suggests the important role of KISS1 in nasopharyngeal carcinoma, but whether the role of KISS1R in nasopharyngeal carcinoma is remain to be further explored. KISS1 or KISS1R is likely to be an independent prognostic factor in certain cancer types. The difference between the proliferation ability of 5-8F-KISS1 cells and 5-8F-1R-KISS1 cells indicated that the *KISS1* gene may work through another receptor or pathway in NPC cells. Through transcriptome sequencing, we found a series of gene expression changes in cells overexpressing *KISS1*, these genes were involved in cell cycle, signal transduction, biosynthesis metabolism and transcriptional regulation. By RT-PCR and western blot analysis, we found that the LKB1 and phosphorylation of the LKB1 was significantly increased and the expression of AMPKα was subsequently increased in 5-8F-KISS1 cells and 5-8F-1R-KISS1 cells. Moreover, it has been reported that LKB1/AMPK inhibits tumor initiation and progression by arresting the cell cycle in the G1 phase and by promoting cell apoptosis (30, 31). Therefore, overexpression of KISS1 is not only the underlying pathological cause of dysregulation of LKB1, but it is also related to the proliferation of nasopharyngeal carcinoma *via* activated LKB1/AMPK pathway. These results suggest a sufficient and necessary role of activated LKB1/AMPK pathway in the proliferation of nasopharyngeal carcinoma, which may be primed by overexpression of KISS1/KISS1R.

Taken together, the current study illustrates a molecular and signal pathway basis for better understanding of how KISS1/KISS1R alters LKB1 activation and thereby activates the AMPK signaling pathway, ultimately inhibits the proliferation of nasopharyngeal carcinoma. In this regard, the expression of KISS1 may serve as an indicator for diagnosis of NPC in clinical practice, or for preventing its development. In the future, additional experiments will be required to explore the role of KISS1 and KISS1R in other cancers and to determine the exact mechanism of their function.

## Data Availability Statement

The datasets presented in this study can be found in online repositories. The names of the repository/repositories and accession number(s) can be found here https://ngdc.cncb.ac.cn/gsa-human HRA001626.

## Ethics Statement

The studies involving human participants were reviewed and approved by The Ethics Committee of the First Affiliated Hospital of Bengbu Medical College. The patients/participants provided their written informed consent to participate in this study. Written informed consent was obtained from the individual(s) for the publication of any potentially identifiable images or data included in this article.

## Author Contributions

TL and YT designed the studies, conducted most of the experiments and data analysis, and wrote the draft manuscript. YW, ZC, ZH, and XW conducted some of the molecular and animal experiments. YZ, and HJ were involved in the overall design of the study and the revision of the final manuscript. HJ and YZ were involved in the overall design of the project, individual experiments, data analysis, and the writing of the final manuscript. All authors contributed to the article and approved the submitted version.

## Funding

This work was supported by the Joint Science and Technology Project of Bengbu Medical College (grants BYLK201805).

## Conflict of Interest

The authors declare that the research was conducted in the absence of any commercial or financial relationships that could be construed as a potential conflict of interest.

## Publisher’s Note

All claims expressed in this article are solely those of the authors and do not necessarily represent those of their affiliated organizations, or those of the publisher, the editors and the reviewers. Any product that may be evaluated in this article, or claim that may be made by its manufacturer, is not guaranteed or endorsed by the publisher.

## Supplementary Material

Supplementary Table 1 Primes used for RT-qPCR.
