# Supplementary material for: Kiss1 Inhibits the Proliferation of Nasopharyngeal Carcinoma Cells Via Activation of the LKB1/AMPK Pathway
Source: Front Oncol. 2022 Jan 18;11:724251. doi: 10.3389/fonc.2021.724251 (PMC8804215; doi:10.3389/fonc.2021.724251)
Supplement: Supplementary Table 1 — Primes used for RT-qPCR. [file Table_1.docx]

**Table S1. Primes used for RT-qPCR.**

| **Gene** | **Forward primers** | **Reverse primers** |
| --- | --- | --- |
| KISS1 | AGCCGCCAGATCCCCGCA | GCCGAAGGAGTTCCAGTTGTAGTT |
| KISS1R | CTCTGACCGCCATGAGTGT | AGAGCCTACCCAGATGCTGA |
| CCND1 | AACAGATCATCCGCAAACAC | GGGTCACACTTGATCACTCT |
| CCND3 | CTCCTACTTCCAGTGCGTG | GGTAGCGATCCAGGTAGTTC |
| CCNE1 | CCAGATGAAGAAATGGCCAAA | GGTAAACCCGGTCATCATCT |
| CCNE2 | CTGGTCTGGCGAGGTTTT | TGCTGCTTAGCTTGTAAACG |
| CCNA1 | GACCAGAGGGGACGTGT | CAGTGCATTGCTTCAGACTC |
| CCNA2 | TTGACTTAGCTGCTCCAACA | TTGAGGTATGGGTCAGCATC |
| CCNB1 | CAACTTGAGGAAGAGCAAGC | TCTCCTGCAACAACCTGAAT |
| CCNB2 | GGGATTACTGCTCTGCTCTT | TGATGCTCGCCTTAAGAAGT |
| CDKN1A | AGACTCTCAGGGTCGAAAAC | TAGGGCTTCCTCTTGGAGAA |
| CDKN1B | CTAACTCTGAGGACACGCAT | AGGTCGCTTCCTTATTCCTG |
| CDK1 | CTGGCTCTTGGAAATTGAGC | GGCTACCACTTGACCTGTAG |
| CDK2 | AAGCCAGAAACAAGTTGACG | ATGACATCCAGCAGCGTG |
| CDK4 | TGTTCCCTCTTGTCCCTTTC | AAGGTTTTGCAGGAAAGTCC |
| CDK6 | TGATTACCTGCTCCGCGA | GTCCAGAATCATTGCACCTG |
| CHK1 | GACAGTCCGCCGAGGT | TGAACTTCTCCATAGGCACC |
| CHK2 | GAAGCTACCTGCAAGCTCTA | CTTGGAGTGCCCAAAATCAG |
| LKB1 | GGTTCCGGAAGAAACATCCT | CAGACTCACCGGGCAC |
| CAMKK1 | ATCCTCCCACTCTCACTGTC | TTCTAGCCCCGACTAACCTT |
| CAMKK2 | ACAAGAGAAGCACATCGGAA | AAACAGCCAAAAGGGAATCG |
| AMPKα1 | TGAAAAAGAAAGTCGGCGTC | TTCATGTGTGCATCAAGCAG |
| AMPKα2 | CCTTCGGCAAAGTGAAGAAAT | CCAACAACATCTAAACTGCGA |
| GAPDH | AATCCCATCACCATCTTCCA | GGCGGAGATGACCCTT |
